# The genome sequence of the Pale November moth, *Epirrita christyi *(Allen, 1906)

**DOI:** 10.12688/wellcomeopenres.23060.1

**Published:** 2024-09-20

**Authors:** Douglas Boyes, Liam M. Crowley, Clare Boyes, University of Oxford and Wytham Woods Genome Acquisition Lab, Darwin Tree of Life Barcoding collective, Wellcome Sanger Institute Tree of Life Management, Samples and Laboratory team, Wellcome Sanger Institute Scientific Operations: Sequencing Operations, Wellcome Sanger Institute Tree of Life Core Informatics team, Tree of Life Core Informatics collective, Darwin Tree of Life Consortium

**Affiliations:** 1 UK Centre for Ecology & Hydrology, Wallingford, England, UK; 2 University of Oxford, Oxford, England, UK; 3 Independent researcher, Welshpool, Wales, UK

**Keywords:** Epirrita christyi, Pale November moth, genome sequence, chromosomal, Lepidoptera

## Abstract

We present a genome assembly from an individual female Pale November moth, *Epirrita christyi* (Arthropoda; Insecta; Lepidoptera; Geometridae). The genome sequence has a total length of 474.20 megabases. Most of the assembly is scaffolded into 31 chromosomal pseudomolecules, including the Z sex chromosome. The mitochondrial genome has also been assembled and is 15.99 kilobases in length. Gene annotation of this assembly on Ensembl identified 16,983 protein-coding genes.

## Species taxonomy

Eukaryota; Opisthokonta; Metazoa; Eumetazoa; Bilateria; Protostomia; Ecdysozoa; Panarthropoda; Arthropoda; Mandibulata; Pancrustacea; Hexapoda; Insecta; Dicondylia; Pterygota; Neoptera; Endopterygota; Amphiesmenoptera; Lepidoptera; Glossata; Neolepidoptera; Heteroneura; Ditrysia; Obtectomera; Geometroidea; Geometridae; Larentiinae; *Epirrita*; *Epirrita christyi* (Allen, 1906) (NCBI:txid247947).

## Background

The Pale November Moth ( *Epirrita christyi*) is a moth in the family Geometridae. It is common and widespread in Britain, although not often found in Ireland, and has declined significantly since 1970 ( Randle *et al.*, 2019). It is present throughout Central and Northern Europe ( GBIF Secretariat, 2024).

The adult moth is difficult to identify as there are four similar *Epirrita* species in the UK and it is therefore likely to be under-recorded. The forewing background can range from dark grey to light grey and can be plain or banded. Colouration and patterning do not indicate species as there is significant variation within and between species. Morphological features are required for correct identification although this only applies to males: females cannot reliably be determined. The moth occurs in mature woodland and can be found by tapping the lower branches of trees. It also flies to light and is on the wing between late September and November. The egg is laid on a twig where it overwinters, hatching in late April, before pupating underground. Larval foodplants are deciduous trees and includes elms, birches, hawthorns and sallows, although there is some debate about the exact requirements because of the identification difficulties ( Waring *et al.*, 2017).

The genome of *Epirrita christyi* was sequenced as part of the Darwin Tree of Life Project, a collaborative effort to sequence all named eukaryotic species in the Atlantic Archipelago of Britain and Ireland. Here we present a chromosomally complete genome sequence for *Epirrita christyi* based on one adult specimen from Wytham Woods, Oxfordshire, UK.

## Genome sequence report

**Figure 1.**
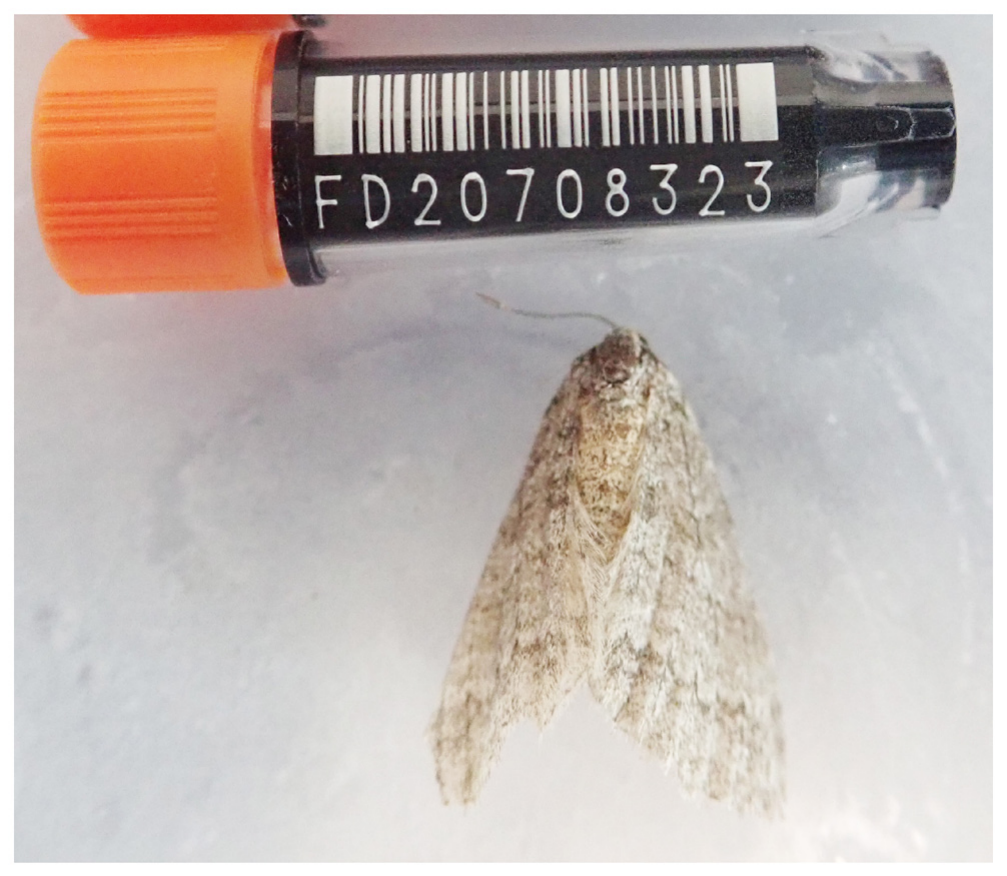
Photograph of the *Epirrita christyi* (ilEpiChri1) specimen used for genome sequencing.

The genome of an adult female *Epirrita christyi* ( Figure 1) was sequenced using Pacific Biosciences single-molecule HiFi long reads, generating a total of 23.55 Gb (gigabases) from 2.22 million reads, providing approximately 48-fold coverage. Primary assembly contigs were scaffolded with chromosome conformation Hi-C data, which produced 97.18 Gb from 643.55 million reads, yielding an approximate coverage of 205-fold. Specimen and sequencing information is summarised in Table 1.

**Table 1.** Specimen and sequencing data for *Epirrita christyi*.

Project information			
**Study title**	*Epirrita christyi* (pale November moth)		
**Umbrella BioProject**	PRJEB60639		
**Species**	*Epirrita christyi*		
**BioSample**	SAMEA8603217		
**NCBI taxonomy ID**	247947		
Specimen information			
**Technology**	**ToLID**	**BioSample accession**	**Organism part**
**PacBio long read sequencing**	ilEpiChri1	SAMEA8603789	Abdomen
**Hi-C sequencing**	ilEpiChri1	SAMEA8603785	Head
**RNA sequencing**	ilEpiChri3	SAMEA113425986	Whole organism
Sequencing information			
**Platform**	**Run accession**	**Read count**	**Base count (Gb)**
**Hi-C Illumina NovaSeq 6000**	ERR11040168	6.44e+08	97.18
**PacBio Sequel IIe**	ERR11029660	2.22e+06	23.55
**RNA Illumina NovaSeq X**	ERR12765138	7.07e+07	10.68

**Figure 2.**
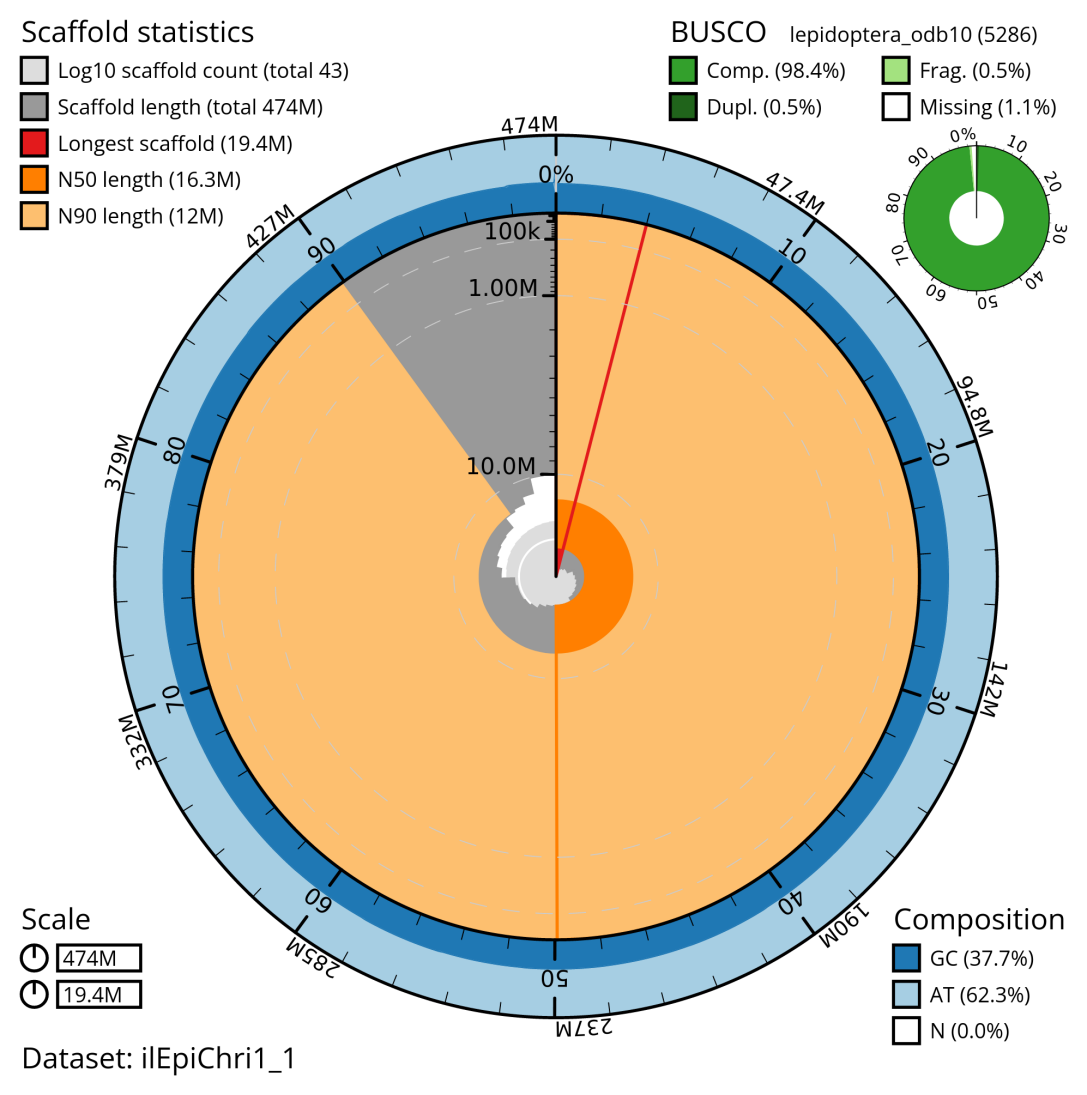
Genome assembly of *Epirrita christyi*, ilEpiChri1.1: metrics. The BlobToolKit snail plot shows N50 metrics and BUSCO gene completeness. The main plot is divided into 1,000 size-ordered bins around the circumference with each bin representing 0.1% of the 474,169,335 bp assembly. The distribution of scaffold lengths is shown in dark grey with the plot radius scaled to the longest scaffold present in the assembly (19,350,054 bp, shown in red). Orange and pale-orange arcs show the N50 and N90 scaffold lengths (16,313,595 and 11,999,344 bp), respectively. The pale grey spiral shows the cumulative scaffold count on a log scale with white scale lines showing successive orders of magnitude. The blue and pale-blue area around the outside of the plot shows the distribution of GC, AT and N percentages in the same bins as the inner plot. A summary of complete, fragmented, duplicated and missing BUSCO genes in the lepidoptera_odb10 set is shown in the top right. An interactive version of this figure is available at https://blobtoolkit.genomehubs.org/view/ilEpiChri1_1/dataset/ilEpiChri1_1/snail.

**Figure 3.**
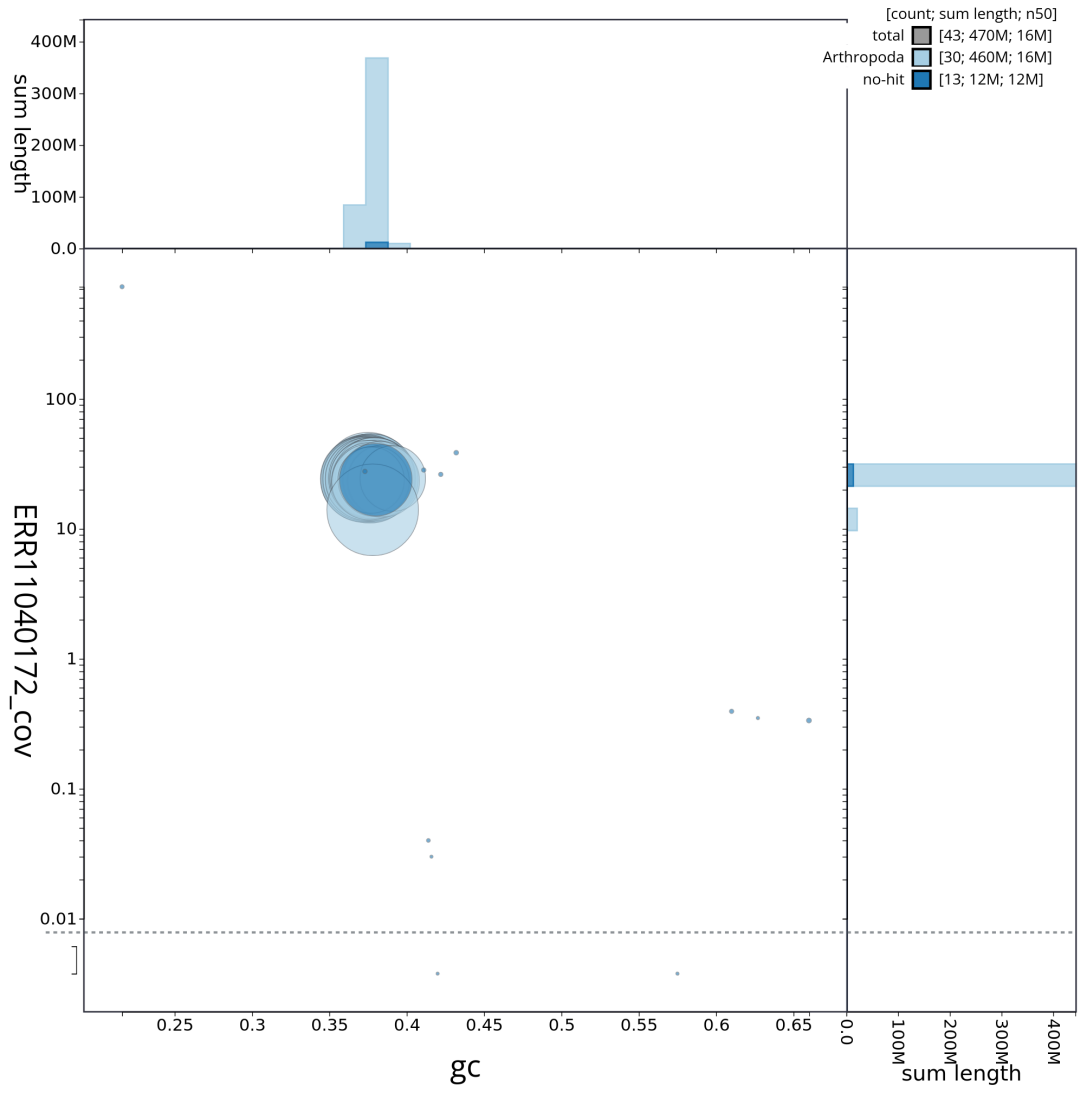
Genome assembly of *Epirrita christyi*, ilEpiChri1.1: Blob plot of base coverage against GC proportion for sequences in the assembly. Sequences are coloured by phylum. Circles are sized in proportion to sequence length. Histograms show the distribution of sequence length sum along each axis. An interactive version of this figure is available at https://blobtoolkit.genomehubs.org/view/ilEpiChri1_1/dataset/ilEpiChri1_1/blob.

**Figure 4.**
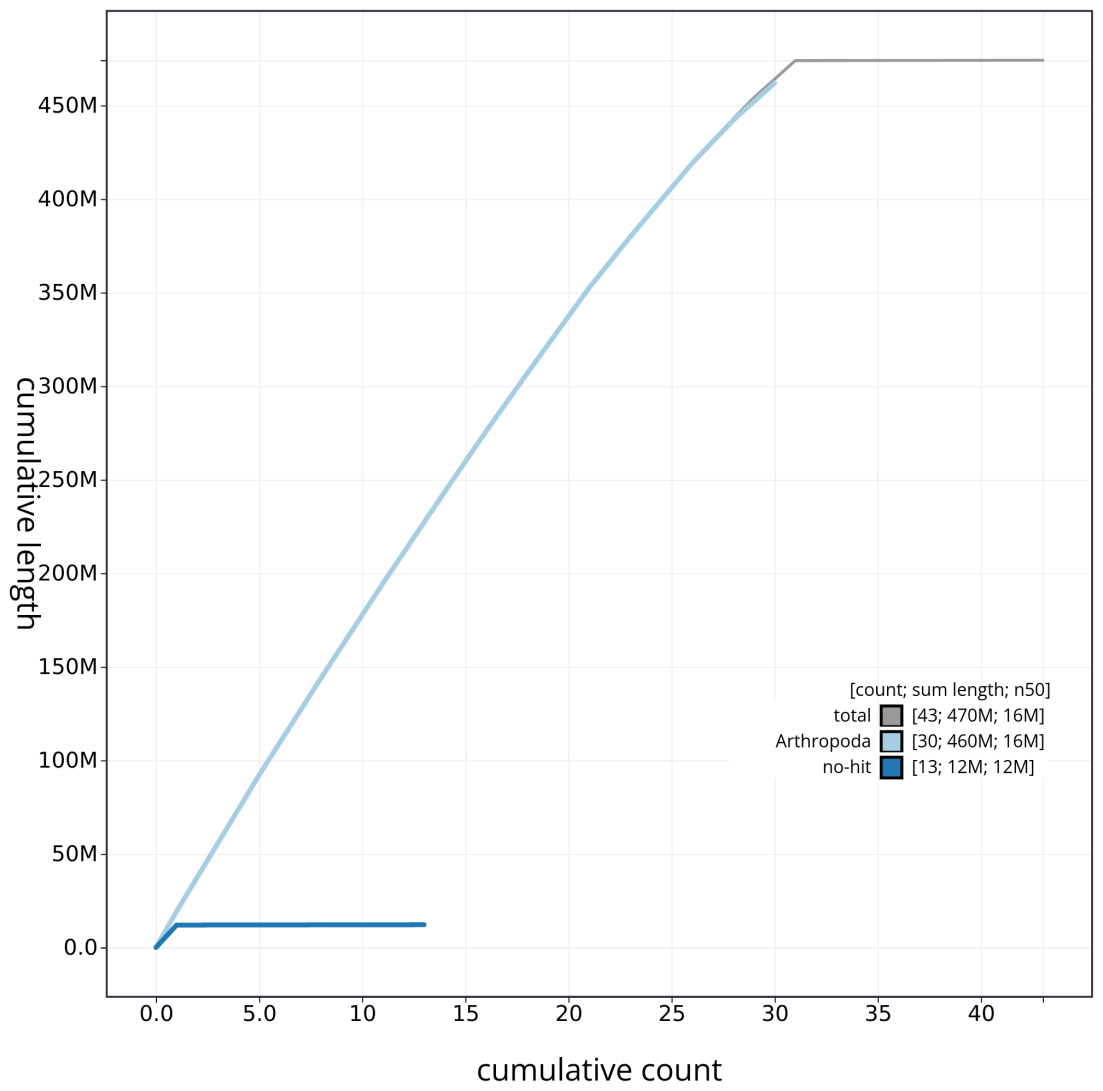
Genome assembly of *Epirrita christyi* ilEpiChri1.1: BlobToolKit cumulative sequence plot. The grey line shows cumulative length for all sequences. Coloured lines show cumulative lengths of sequences assigned to each phylum using the buscogenes taxrule. An interactive version of this figure is available at https://blobtoolkit.genomehubs.org/view/ilEpiChri1_1/dataset/ilEpiChri1_1/cumulative.

**Figure 5.**
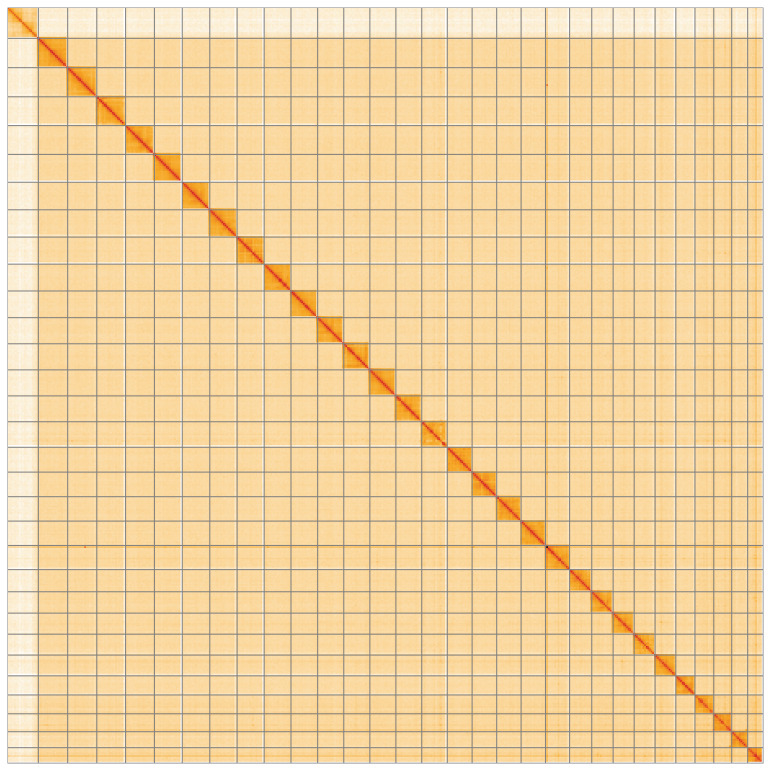
Genome assembly of *Epirrita christyi* ilEpiChri1.1: Hi-C contact map of the ilEpiChri1.1 assembly, visualised using HiGlass. Chromosomes are shown in order of size from left to right and top to bottom. An interactive version of this figure may be viewed at https://genome-note-higlass.tol.sanger.ac.uk/l/?d=cXBs9OpPQsu7CmKGlsRvOA.

Manual assembly curation corrected 48 missing joins or mis-joins and 19 haplotypic duplications, reducing the assembly length by 1.41% and the scaffold number by 30.65%, and increasing the scaffold N50 by 0.46%. The final assembly has a total length of 474.20 Mb in 42 sequence scaffolds with a scaffold N50 of 16.3 Mb ( Table 2). The snail plot in Figure 2 provides a summary of the assembly statistics, while the distribution of assembly scaffolds on GC proportion and coverage is shown in Figure 3. The cumulative assembly plot in Figure 4 shows curves for subsets of scaffolds assigned to different phyla. Most (99.96%) of the assembly sequence was assigned to 31 chromosomal-level scaffolds, representing 30 autosomes and the Z sex chromosome. Chromosome-scale scaffolds confirmed by the Hi-C data are named in order of size ( Figure 5; Table 3). The Z chromosome was identified by coverage, and by alignment to *Gymnoscelis rufifasciata* (GCA_929108375.1) ( Boyes *et al.*, 2022), *Electrophaes corylata* (GCA_947095575.1) ( Boyes *et al.*, 2023b), *Thera britannica* (GCA_939531255.2) ( Boyes *et al.*, 2023c) and *Anticlea derivata* (GCA_947579855.1) ( Boyes *et al.*, 2023a). While the Z chromosome has approximately half read coverage, no W chromosome could be identified, and the specimen is likely to be a ZO female. While not fully phased, the assembly deposited is of one haplotype. Contigs corresponding to the second haplotype have also been deposited. The mitochondrial genome was also assembled and can be found as a contig within the multifasta file of the genome submission.

**Table 2.** Genome assembly data for *Epirrita christyi*, ilEpiChri1.1.

Genome assembly		
Assembly name	ilEpiChri1.1	
Assembly accession	GCA_951392215.1	
*Accession of alternate haplotype*	*GCA_949802565.1*	
Span (Mb)	474.20	
Number of contigs	142	
Contig N50 length (Mb)	6.1	
Number of scaffolds	42	
Scaffold N50 length (Mb)	16.3	
Longest scaffold (Mb)	19.35	
Assembly metrics *		*Benchmark*
Consensus quality (QV)	64.9	*≥ 50*
*k*-mer completeness	100.0%	*≥ 95%*
BUSCO **	C:98.4%[S:97.9%,D:0.5%], F:0.5%,M:1.1%,n:5,286	*C ≥ 95%*
Percentage of assembly mapped to chromosomes	99.96%	*≥ 95%*
Sex chromosomes	Z	*localised homologous pairs*
Organelles	Mitochondrial genome: 15.99 kb	*complete single alleles*
Genome annotation of assembly GCA_951392215.1 at Ensembl		
Number of protein-coding genes	16,983	
Number of gene transcripts	17,160	

* Assembly metric benchmarks are adapted from column VGP-2020 of “Table 1: Proposed standards and metrics for defining genome assembly quality” from Rhie *et al.* (2021). ** BUSCO scores based on the lepidoptera_odb10 BUSCO set using version 5.3.2. C = complete [S = single copy, D = duplicated], F = fragmented, M = missing, n = number of orthologues in comparison. A full set of BUSCO scores is available at https://blobtoolkit.genomehubs.org/view/ilEpiChri1_1/dataset/ilEpiChri1_1/busco.

**Table 3.** Chromosomal pseudomolecules in the genome assembly of *Epirrita christyi*, ilEpiChri1.

INSDC accession	Name	Length (Mb)	GC%
OX595834.1	1	18.39	37.5
OX595835.1	2	18.27	37.5
OX595836.1	3	18.06	37.5
OX595837.1	4	18.05	37.5
OX595838.1	5	17.46	37.5
OX595839.1	6	17.29	37.5
OX595840.1	7	17.12	37.5
OX595841.1	8	16.97	37.0
OX595842.1	9	16.84	37.5
OX595843.1	10	16.74	37.0
OX595844.1	11	16.37	37.5
OX595845.1	12	16.36	37.5
OX595846.1	13	16.31	37.5
OX595847.1	14	16.22	37.5
OX595848.1	15	16.0	38.0
OX595849.1	16	15.59	37.5
OX595850.1	17	15.41	37.5
OX595851.1	18	15.3	37.5
OX595852.1	19	15.3	38.0
OX595853.1	20	15.14	38.0
OX595854.1	21	13.9	38.0
OX595855.1	22	13.4	37.5
OX595856.1	23	13.21	37.5
OX595857.1	24	13.1	37.5
OX595858.1	25	13.0	38.5
OX595859.1	26	12.0	38.0
OX595860.1	27	11.82	38.5
OX595861.1	28	11.24	38.0
OX595862.1	29	10.01	37.5
OX595863.1	30	9.76	39.0
OX595833.1	Z	19.35	38.0
OX595864.1	MT	0.02	21.5

The estimated Quality Value (QV) of the final assembly is 64.9 with *k*-mer completeness of 100.0%, and the assembly has a BUSCO v5.3.2 completeness of 98.4% (single = 97.9%, duplicated = 0.5%), using the lepidoptera_odb10 reference set ( *n* = 5286).

Metadata for specimens, BOLD barcode results, spectra estimates, sequencing runs, contaminants and pre-curation assembly statistics are given at https://links.tol.sanger.ac.uk/species/247947.

### Genome annotation report

The *Epirrita christyi* genome assembly (GCA_951392215.1) was annotated at the European Bioinformatics Institute (EBI) on Ensembl Rapid Release. The resulting annotation includes 17,160 transcribed mRNAs from 16,983 protein-coding genes ( Table 2; https://rapid.ensembl.org/Epirrita_christyi_GCA_951392215.1/Info/Index). The average transcript length is 7,844.37. There are 1.01 coding transcripts per gene and 5.84 exons per transcript.

## Methods

### Sample acquisition and barcoding

An adult female *Epirrita christyi* (specimen ID Ox000994, ToLID ilEpiChri1) was collected from Wytham Woods, Oxfordshire (biological vice-county Berkshire), UK (latitude 51.77, longitude –1.34) on 2020-11-21 using a light trap. The specimen was collected and identified by Douglas Boyes (University of Oxford) and preserved on dry ice. This specimen was used for PacBio HiFi and Illumina Hi-C sequencing.

The specimen used for RNA sequencing (specimen ID Ox003249, ToLID ilEpiChri3) was an adult specimen collected from the same location on 2022-10-20, using a light trap. The specimen was collected and identified by Liam Crowley (University of Oxford) and preserved on dry ice.

The initial identification was verified by an additional DNA barcoding process according to the framework developed by Twyford *et al*. (2024). A small sample was dissected from the specimens and stored in ethanol, while the remaining parts of the specimen were shipped on dry ice to the Wellcome Sanger Institute (WSI). The tissue was lysed, the COI marker region was amplified by PCR, and amplicons were sequenced and compared to the BOLD database, confirming the species identification ( Crowley *et al.*, 2023). Following whole genome sequence generation, the relevant DNA barcode region was also used alongside the initial barcoding data for sample tracking at the WSI ( Twyford *et al.*, 2024). The standard operating procedures for Darwin Tree of Life barcoding have been deposited on protocols.io ( Beasley *et al.*, 2023).

### Nucleic acid extraction

The workflow for high molecular weight (HMW) DNA extraction at the Wellcome Sanger Institute (WSI) Tree of Life Core Laboratory includes a sequence of core procedures: sample preparation and homogenisation, DNA extraction, fragmentation and purification. Detailed protocols are available on protocols.io ( Denton *et al.*, 2023b). In sample preparation, the ilEpiChri1 sample was weighed and dissected on dry ice ( Jay *et al.*, 2023). Tissue from the abdomen was homogenised using a PowerMasher II tissue disruptor ( Denton *et al.*, 2023a). HMW DNA was extracted using the Automated MagAttract v1 protocol ( Sheerin *et al.*, 2023). DNA was sheared into an average fragment size of 12–20 kb in a Megaruptor 3 system ( Todorovic *et al.*, 2023). Sheared DNA was purified by solid-phase reversible immobilisation, using AMPure PB beads to eliminate shorter fragments and concentrate the DNA ( Strickland *et al.*, 2023). The concentration of the sheared and purified DNA was assessed using a Nanodrop spectrophotometer and Qubit Fluorometer using the Qubit dsDNA High Sensitivity Assay kit. Fragment size distribution was evaluated by running the sample on the FemtoPulse system.

RNA was extracted from whole organism tissue of ilEpiChri3 in the Tree of Life Laboratory at the WSI using the RNA Extraction: Automated MagMax™ *mir*Vana protocol ( do Amaral *et al.*, 2023). The RNA concentration was assessed using a Nanodrop spectrophotometer and a Qubit Fluorometer using the Qubit RNA Broad-Range Assay kit. Analysis of the integrity of the RNA was done using the Agilent RNA 6000 Pico Kit and Eukaryotic Total RNA assay.

### Library preparation and sequencing

Pacific Biosciences HiFi circular consensus DNA sequencing libraries were constructed according to the manufacturers’ instructions. Poly(A) RNA-Seq libraries were constructed using the NEB Ultra II RNA Library Prep kit. DNA and RNA sequencing was performed by the Scientific Operations core at the WSI on Pacific Biosciences Sequel IIe (HiFi) and Illumina NovaSeq X (RNA-Seq) instruments.

Hi-C data were generated from the head tissue of the ilEpiChri1 sample, using the Arima-HiC v2 kit. In brief, frozen tissue (–80°C) was fixed, and the DNA crosslinked using a TC buffer containing formaldehyde. The crosslinked DNA was then digested using a restriction enzyme master mix. The 5’-overhangs were then filled in and labelled with a biotinylated nucleotide and proximally ligated. The biotinylated DNA construct was fragmented to a fragment size of 400 to 600 bp using a Covaris E220 sonicator. The DNA was then enriched, barcoded, and amplified using the NEBNext Ultra II DNA Library Prep Kit, following manufacturers’ instructions. The Hi-C sequencing was performed using paired-end sequencing with a read length of 150 bp on an Illumina NovaSeq 6000 instrument.

### Genome assembly, curation and evaluation

***Assembly***

The HiFi reads were first assembled using Hifiasm ( Cheng *et al.*, 2021) with the --primary option. Haplotypic duplications were identified and removed using purge_dups ( Guan *et al.*, 2020). The Hi-C reads were mapped to the primary contigs using bwa-mem2 ( Vasimuddin *et al.*, 2019). The contigs were further scaffolded using the provided Hi-C data ( Rao *et al.*, 2014) in YaHS ( Zhou *et al.*, 2023) using the --break option. The scaffolded assemblies were evaluated using Gfastats ( Formenti *et al.*, 2022), BUSCO ( Manni *et al.*, 2021) and MERQURY.FK ( Rhie *et al.*, 2020).

The mitochondrial genome was assembled using MitoHiFi ( Uliano-Silva *et al.*, 2023), which runs MitoFinder ( Allio *et al.*, 2020) and uses these annotations to select the final mitochondrial contig and to ensure the general quality of the sequence.

***Assembly curation***

The assembly was decontaminated using the Assembly Screen for Cobionts and Contaminants (ASCC) pipeline (article in preparation). Manual curation was primarily conducted using PretextView ( Harry, 2022), with additional insights provided by JBrowse2 ( Diesh *et al.*, 2023) and HiGlass ( Kerpedjiev *et al.*, 2018). Scaffolds were visually inspected and corrected as described by Howe *et al*. (2021). Any identified contamination, missed joins, and mis-joins were corrected, and duplicate sequences were tagged and removed. Sex chromosomes were identified by synteny analysis. The curation process is documented at https://gitlab.com/wtsi-grit/rapid-curation (article in preparation).

***Evaluation of the final assembly***

A Hi-C map for the final assembly was produced using bwa-mem2 ( Vasimuddin *et al.*, 2019) in the Cooler file format ( Abdennur & Mirny, 2020). To assess the assembly metrics, the *k*-mer completeness and QV consensus quality values were calculated in Merqury ( Rhie *et al.*, 2020). This work was done using the “sanger-tol/readmapping” ( Surana *et al.*, 2023a) and “sanger-tol/genomenote” ( Surana *et al.*, 2023b) pipelines. The genome readmapping pipelines were developed using the nf-core tooling ( Ewels *et al.*, 2020), use MultiQC ( Ewels *et al.*, 2016), and make extensive use of the Conda package manager, the Bioconda initiative ( Grüning *et al.*, 2018), the Biocontainers infrastructure ( da Veiga Leprevost *et al.*, 2017), and the Docker ( Merkel, 2014) and Singularity ( Kurtzer *et al.*, 2017) containerisation solutions. The genome was also analysed within the BlobToolKit environment ( Challis *et al.*, 2020) and BUSCO scores ( Manni *et al.*, 2021) were calculated.

Table 4 contains a list of relevant software tool versions and sources.

**Table 4.** Software tools: versions and sources.

Software tool	Version	Source
BlobToolKit	4.2.1	https://github.com/blobtoolkit/blobtoolkit
BUSCO	5.3.2	https://gitlab.com/ezlab/busco
bwa-mem2	2.2.1	https://github.com/bwa-mem2/bwa-mem2
Cooler	0.8.11	https://github.com/open2c/cooler
Gfastats	1.3.6	https://github.com/vgl-hub/gfastats
Hifiasm	0.16.1-r375	https://github.com/chhylp123/hifiasm
HiGlass	1.11.6	https://github.com/higlass/higlass
Merqury.FK	d00d98157618f4e8d1a9190026b19b471055b22e	https://github.com/thegenemyers/MERQURY.FK
MitoHiFi	2	https://github.com/marcelauliano/MitoHiFi
PretextView	0.2	https://github.com/wtsi-hpag/PretextView
purge_dups	1.2.3	https://github.com/dfguan/purge_dups
sanger-tol/genomenote	v1.0	https://github.com/sanger-tol/genomenote
sanger-tol/readmapping	1.1.0	https://github.com/sanger-tol/readmapping/tree/1.1.0
Singularity	3.9.0	https://github.com/sylabs/singularity
YaHS	1.2a	https://github.com/c-zhou/yahs

### Genome annotation

The BRAKER2 pipeline ( Brůna *et al.*, 2021) was used in the default protein mode to generate annotation for the *Epirrita christyi* assembly (GCA_951392215.1) in Ensembl Rapid Release at the EBI.

### Wellcome Sanger Institute – Legal and Governance

The materials that have contributed to this genome note have been supplied by a Darwin Tree of Life Partner. The submission of materials by a Darwin Tree of Life Partner is subject to the **‘Darwin Tree of Life Project Sampling Code of Practice’**, which can be found in full on the Darwin Tree of Life website here. By agreeing with and signing up to the Sampling Code of Practice, the Darwin Tree of Life Partner agrees they will meet the legal and ethical requirements and standards set out within this document in respect of all samples acquired for, and supplied to, the Darwin Tree of Life Project.

Further, the Wellcome Sanger Institute employs a process whereby due diligence is carried out proportionate to the nature of the materials themselves, and the circumstances under which they have been/are to be collected and provided for use. The purpose of this is to address and mitigate any potential legal and/or ethical implications of receipt and use of the materials as part of the research project, and to ensure that in doing so we align with best practice wherever possible. The overarching areas of consideration are:

- Ethical review of provenance and sourcing of the material
- Legality of collection, transfer and use (national and international)

Each transfer of samples is further undertaken according to a Research Collaboration Agreement or Material Transfer Agreement entered into by the Darwin Tree of Life Partner, Genome Research Limited (operating as the Wellcome Sanger Institute), and in some circumstances other Darwin Tree of Life collaborators.

## Data Availability

European Nucleotide Archive: *Epirrita christyi* (pale November moth). Accession number PRJEB60639; https://identifiers.org/ena.embl/PRJEB60639 ( Wellcome Sanger Institute, 2023). The genome sequence is released openly for reuse. The *Epirrita christyi* genome sequencing initiative is part of the Darwin Tree of Life (DToL) project. All raw sequence data and the assembly have been deposited in INSDC databases. Raw data and assembly accession identifiers are reported in Table 1 and Table 2.

## Author information

Members of the University of Oxford and Wytham Woods Genome Acquisition Lab are listed here: https://doi.org/10.5281/zenodo.12157525.

Members of the Darwin Tree of Life Barcoding collective are listed here: https://doi.org/10.5281/zenodo.12158331

Members of the Wellcome Sanger Institute Tree of Life Management, Samples and Laboratory team are listed here: https://doi.org/10.5281/zenodo.12162482.

Members of Wellcome Sanger Institute Scientific Operations: Sequencing Operations are listed here: https://doi.org/10.5281/zenodo.12165051.

Members of the Wellcome Sanger Institute Tree of Life Core Informatics team are listed here: https://doi.org/10.5281/zenodo.12160324.

Members of the Tree of Life Core Informatics collective are listed here: https://doi.org/10.5281/zenodo.12205391.

Members of the Darwin Tree of Life Consortium are listed here: https://doi.org/10.5281/zenodo.4783558.
