# Supplementary material for: The genome sequence of the Pale November moth, Epirrita christyi (Allen, 1906)
Source: Wellcome Open Res. 2024 Sep 20;9:540. [Version 1] doi: 10.12688/wellcomeopenres.23060.1 (PMC11549545; doi:10.12688/wellcomeopenres.23060.1)
Supplement: Peer review history

# Reviewer report: Reviewer response for version 1

**DOI:** 10.21956/wellcomeopenres.25396.r109742

**Published:** 2024-11-18

**Authors:** Shruti Iyer

**Affiliations:** 1 Cold Spring Harbor Laboratory, Cold Spring Harbor, New York, USA

Review of “The genome sequence of the Pale November moth, *Epirrita christyi *(Allen, 1906)”

 The authors have done a great job generating a good quality genome, while also including the mitochondrial genome and annotating the assembled genome.

 In terms of the text, I think the manuscript would benefit with an expansion of the background. *Epirrita christyi *seems difficult to accurately identify. While the authors touch upon this difficulty, especially when identifying adult females, they do not elaborate on how they were confident of the sample they collected. Confirmation of species genomically is touched upon later, but addressing this earlier in the text is recommended.

 With respect to the figures, I think the color scheme needs to be modified in some of them to improve visibility.

- Figure 2: using shades of green for the BUSCO plot make it difficult to discern between the categories, especially since this part of the figure isn’t interactive.
- Figure 3: the colors for total (gray) and arthropoda (light blue) are too similar to distinguish between them. Changing the color scheme will help with better visualization.

Are sufficient details of methods and materials provided to allow replication by others?

Yes

Is the rationale for creating the dataset(s) clearly described?

Yes

Are the datasets clearly presented in a useable and accessible format?

Yes

Are the protocols appropriate and is the work technically sound?

Yes

Reviewer Expertise:

genetics, genomics, sequencing technologies, technology development, targeted sequencing.

I confirm that I have read this submission and believe that I have an appropriate level of expertise to confirm that it is of an acceptable scientific standard.

# Reviewer report: Reviewer response for version 1

**DOI:** 10.21956/wellcomeopenres.25396.r109737

**Published:** 2024-11-08

**Authors:** Arun Arumugaperumal

**Affiliations:** 1 Department of Biotechnology, Rajalakshmi Engineering College, Tamilnadu, Thandalam, Chennai, 602105, India

This is the first published article on the genome sequence of the Pale November moth *Epirrita christyi*. The assembly was of length 474.2 Mb. The mitogenome was also assembled and of size 15.99 kb. The assembly had allowed identification of 16,983 genes. Long read sequencing technology has been used to arrive at a near-complete genome sequence. The methods followed have been clearly explained.

Are sufficient details of methods and materials provided to allow replication by others?

Yes

Is the rationale for creating the dataset(s) clearly described?

Yes

Are the datasets clearly presented in a useable and accessible format?

Yes

Are the protocols appropriate and is the work technically sound?

Yes

Reviewer Expertise:

Bioinformatics, Genomics

I confirm that I have read this submission and believe that I have an appropriate level of expertise to confirm that it is of an acceptable scientific standard.

# Reviewer report: Reviewer response for version 1

**DOI:** 10.21956/wellcomeopenres.25396.r103842

**Published:** 2024-10-18

**Authors:** Ben Raymond, Zoltan Erdos

**Affiliations:** 1 University of Exeter Cornwall Campus (Ringgold ID: 151778), Penryn, England, UK

This is a very high quality genome of a nice little geometrid that is hard to identify from morphological traits.

 I have few comments - I thought the background section could also mention that the larvae in this genus are also hard to separate based on morphology- something that is complicated by the occurence of melanic forms.   The implication that a genome could aid future identifiication could be stated explicitly.

 I wasn't convinced of the value of all the blob plots - eg Fig 4.    This could have been summarized in the text.

 The chromosomal level assembly was particularly impressive..

Are sufficient details of methods and materials provided to allow replication by others?

Yes

Is the rationale for creating the dataset(s) clearly described?

Yes

Are the datasets clearly presented in a useable and accessible format?

Yes

Are the protocols appropriate and is the work technically sound?

Yes

Reviewer Expertise:

Entomology, evolutionary biology, ecology

We confirm that we have read this submission and believe that we have an appropriate level of expertise to confirm that it is of an acceptable scientific standard.
